# The Diagnostic Yield of [^18^F]FDG-PET/CT in a Heterogeneous In-Patient Population with Suspected Infection or Inflammation Is Comparable to Findings in Patients with Classic Fever of Unknown Origin

**DOI:** 10.3390/diagnostics14131420

**Published:** 2024-07-03

**Authors:** Kristian Kimer Becker, Jacob Søholm, Søren Hess

**Affiliations:** 1 Department of Clinical Research, Faculty of Health Sciences, University of Southern Denmark, 5230 Odense, Denmark; kristian.kimer@hotmail.com; 2 IRIS—Imaging Research Initiative Southwest, University Hospital of Southern Denmark, 6700 Esbjerg, Denmark; 3 Department of Infectious Diseases, Odense University Hospital, 5000 Odense, Denmark; jacob.soeholm@rsyd.dk; 4 Department of Nuclear Medicine, Odense University Hospital, 5000 Odense, Denmark

**Keywords:** FDG, PET, PET/CT, FUO, fever of unknown origin, infection

## Abstract

Introduction: Suspected infection or inflammation of unknown origin in in-patients remains challenging. Literature on [^18^F]FDG-PET/CT is abundant in classic fever of unknown origin (FUO), but evidence is complex and may not always reflect clinical reality. This study explores the application of [^18^F]FDG-PET/CT in a diverse clinical population of in-patients with suspected infection not defined by stringent FUO-criteria. Methods: Retrospective chart review of consecutive in-patients who underwent [^18^F]FDG-PET/CT in the workup of suspected infection or inflammation from 1 July 2022 to 31 December 2022 was conducted. We evaluated indications, diagnostic yield, and clinical impact of [^18^F]FDG-PET/CT, and compared the findings of [^18^F]FDG-PET/CT and stand-alone CT. Univariate logistic regression assessed associations between [^18^F]FDG-PET/CT outcome and clinical parameters. Receiver operating characteristic curve (ROC) analysis evaluated diagnostic performance. Results: 77 patients met the inclusion criteria. [^18^F]FDG-PET/CT established a diagnosis in 35% of cases, ruled out focal infection in 26%, and thus was helpful in 61% of patients. It prompted 72 additional examinations resulting in seven incidental diagnoses, including two cancers. Antibiotic treatment was changed in 26% of cases. Regression analysis found white blood cell counts (WBC) associated with true positive outcomes. [^18^F]FDG-PET/CT was compared to stand-alone CT findings, and was concordant in 69% of cases. Conclusions: Results were comparable to findings in more classic FUO. [^18^F]FDG-PET/CT was clinically helpful in 61% of cases but also prompted many additional examinations with relatively few clinically important findings. WBC count was a predictor of true positive outcome. CT and [^18^F]FDG-PET/CT were discordant in 31%, of cases, especially in cases of endocarditis and spondylodiscitis.

## 1. Introduction

Infectious and inflammatory diseases do not always present with obvious symptoms or clinical findings pointing towards their origin. Therefore, imaging plays a key role in the workup to identify and characterise the focal point, and, in difficult-to-diagnose cases, combined positron emission tomography (PET) with 2-deoxy-2-[18F]fluoro-D-glucose tracer (^18^F) and computed tomography ([^18^F]FDG-PET/CT) is increasingly employed. 

[^18^F]FDG-PET/CT combines functional imaging of cellular metabolism with the morphology from CT; activated inflammatory cells accumulate [^18^F]FDG due to their high metabolic activity; and CT helps to identify the precise location of these metabolic cells or directly visualises abscesses or other structural abnormalities associated with infection. However, controversy remains regarding its precise role.

Mounting evidence highlights the general effectiveness of [^18^F]FDG-PET/CT in diagnosing infections and inflammation, as demonstrated by several studies focusing on specific conditions such as vascular graft infection [1,2], infective endocarditis [3,4], staphylococcus aureus bacteraemia [5,6], vasculitis [7], and polymyalgia rheumatica [8]. These conditions (along with several hundred more) may be the underlying cause of fever of unknown origin (FUO), a prolonged, unexplained fever persisting for a certain period despite extensive workup. Given the aforementioned efficacy of [^18^F]FDG-PET/CT in the broad spectrum of potential differential diagnoses, FUO is probably the most well-studied indication for [^18^F]FDG-PET/CT in the infection and inflammation domain [9,10]. However, although FUO represents a precise, academic concept, it is often not an accurate reflection of the clinical reality and the diagnostic challenges posed by inflamed in-patients. The group is heterogeneous, often presenting with non-specific signs and symptoms and with diseases that range from simple self-limiting conditions to metastatic infections or malignancies. Thus, many patients admitted with suspected infections simply do not adhere to the rather stringent criteria, and the findings of [^18^F]FDG-PET/CT in FUO may not directly apply to a broader patient population in actual clinical reality. 

The benefits of introducing [^18^F]FDG-PET/CT in a less stringently selected clinical setting, i.e., in-patients with suspected infection and inflammation, presents a largely unexplored area. With the potential of [^18^F]FDG-PET/CT in FUO and a more widespread availability of combined PET/CT scanners, the use of [^18^F]FDG-PET/CT in actual clinical practice may now be skewed towards earlier, unsubstantiated use. Additionally, with its less-than-ideal specificity, [^18^F]FDG-PET/CT potentially gives rise to higher rates of incidental and false-positive findings, leading to unnecessary supplemental workup or overtreatment of suspected conditions not present, increased healthcare costs, and potential obfuscation of the diagnostic process.

In this retrospective chart review study, we aimed to evaluate the effectiveness of [^18^F]FDG-PET/CT in a heterogeneous and broad clinical population of in-patients with suspected infectious or inflammatory diseases not confined to the classical definitions of FUO. Specifically, we investigated the diagnostic yield of [^18^F]FDG-PET/CT, its impact on antimicrobial treatment, comparative findings of stand-alone CT and [^18^F]FDG-PET/CT, and the consequences of incidental findings. 

## 2. Methods 

### 2.1. Population 

The study was conducted at the Department of Radiology and Nuclear Medicine, University Hospital of Southern Denmark, Esbjerg, a 370-bed secondary-care hospital. Patients who underwent [^18^F]FDG-PET/CT between 1 July 2022 and 31 December 2022 were included. Follow-up was conducted in April 2023. 

Inclusion criteria for the study were: in-patients aged 18 years or older referred for [^18^F]FDG-PET/CT scan in search of an infection source. Exclusion criteria for the study were: outpatients, as these comprise a different population that more often adheres to the stringent definition of FUO, and patients for whom ^18^F-FDG PET/CT scans were performed for non-infectious indications such as oncological staging. Patients could be included more than once if the criteria were met again in a later admission. No fixed preliminary diagnostic protocol was mandatory for inclusion in the study, and, as such, not all patients underwent thorough testing before [^18^F]FDG-PET/CT was performed. 

### 2.2. [^18^F]FDG-PET/CT

[^18^F]FDG-PET/CT was performed on one of two available scanners (Discovery 710 or Discovery MI 5 ring; GE Healthcare, Milwaukee, WI, USA) according to the department standard protocol based on European Association of Nuclear Medicine (EANM) guidelines [11]. 

Scans were preceded by six hours of fasting and discontinued short-acting insulin to ensure sufficient tracer uptake. Blood glucose levels were measured prior to scanning and were required to be under 11 mmol/L. If possible, patients suspected of having infective endocarditis or suspected foci in the heart had to follow a high-fat, low-carbohydrate diet for 12 h, followed by 12 h of prolonged fasting before ^18^F-FDG injection to suppress physiologic glucose uptake in the myocardium and enhance sensitivity. An injection of 4 MBq/kg with lower and upper limits of 200–400 MBq ^18^F-FDG was delivered intravenously, most commonly in the antecubital fossa, unless inaccessible or otherwise infeasible. Patients were scanned 60 min post-injection. PET scans consisted of 2.5 min per bed on Discovery 710 and 1.5 min on Discovery MI. Images were reconstructed in a matrix size of 256 × 256 (pixel size 2.73 mm) using a block sequential regularised expectation maximisation algorithm (Q.Clear, GE Healthcare) with β penalising factor of 500. Attenuation and scatter correction was based on a low-dose helical CT scan at 120 kVp using dose modulation and SmartmA at noise index 75 and pitch 0.984 acquired immediately before the PET, and reconstructed with filtered back-projection and the Q.AC convolution kernel (GE Healthcare, Milwaukee, WI, USA). 

After PET acquisition, a diagnostic CT scan was conducted for radiological diagnostic purposes. An eGFR > 30 mL/min/1.73 m^2^ was required for intravenous contrast. The scan was performed at 120 kVp using dose modulation, SmartmA at noise index 24, pitch 0.984, and reconstructed with the iterative reconstruction algorithm ASIR or ASIR-V at 50% and the Stnd convolution kernel. The PET and CT scan area covered from the vertex of the skull to the mid-thigh level; lower extremities were included when clinically indicated. The results of the [^18^F]FDG-PET/CT scans were separately interpreted by a specialist in nuclear medicine and a specialist in radiology, followed by a joint discussion before a conclusion was made.

### 2.3. Analysis 

Patient data, including demographics, indications, and impact of [^18^F]FDG-PET/CT, were gathered from the patients’ digital medical record based on a thorough evaluation of available data and recorded according to a predefined protocol, cf. Supplementary Tables S1 and S2. 

As reference standard, we used the final diagnosis/diagnoses as a composite of the discharge summary and available information from the patients’ charts. 

[^18^F]FDG-PET/CT was considered “helpful” in two scenarios:

1. (True positive): Establishing a diagnosis corresponding to the final diagnosis by showing FDG uptake in a tissue or an organ deemed responsible for the condition or by CT identifying the pathology, even in the absence of FDG uptake.
2. (True negative): If it was without pathological findings, i.e., no focal or diffuse FDG uptake suggestive of infection or malignancy, and no diagnosis was established during follow-up.

[^18^F]FDG-PET/CT was considered “unhelpful” if the scan did not clinically contribute to the diagnostic process by being inconclusive, not showing focal findings, prompting further examinations into areas that were ultimately not considered responsible for the patient’s condition, or a diagnosis was made through other means. 

To measure the impact of [^18^F]FDG-PET/CT on antimicrobial treatment, we registered whether it led to starting, discontinuing, narrowing, broadening, extending, or shortening antibiotic regimen.

To compare the results of [^18^F]FDG-PET/CT with standalone CT, we had a radiologist, blinded to the PET part, describe only the diagnostic CTs of the true positive cases. The descriptions of the [^18^F]FDG-PET/CT and diagnostic CTs were compared to determine the level of agreement.

### 2.4. Statistical Analysis 

R 4.3.0 was used for statistical analysis. For the subgroup analysis, the χ^2^ test was used to detect differences between patients who had undergone previous imaging and those who had not.

C-reactive protein (CRP) level and white blood cell count (WBC) were analysed with univariate logistic regression as independent variables and PET/CT outcome as the dependent variable.

True positive outcomes were coded as 1 and true negative/unhelpful scans as 0. A *p* < 0.05 was considered statistically significant. A receiver operating characteristic (ROC) curve analysis was conducted to assess the diagnostic performance of CRP and WBC. The area under the curve (AUC) and optimal cut-off were determined for both biomarkers. The optimal cut-off was determined using Youden’s index.

### 2.5. Ethics 

This retrospective chart review study complies with the Declaration of Helsinki and was approved by the review board of the University Hospital of Southern Denmark, Esbjerg. All data were securely stored and processed according to GDPR. This study is regarded as a retrospective observational study, and ethical clearance was exempted under Danish law. 

## 3. Results 

**Figure 1:**
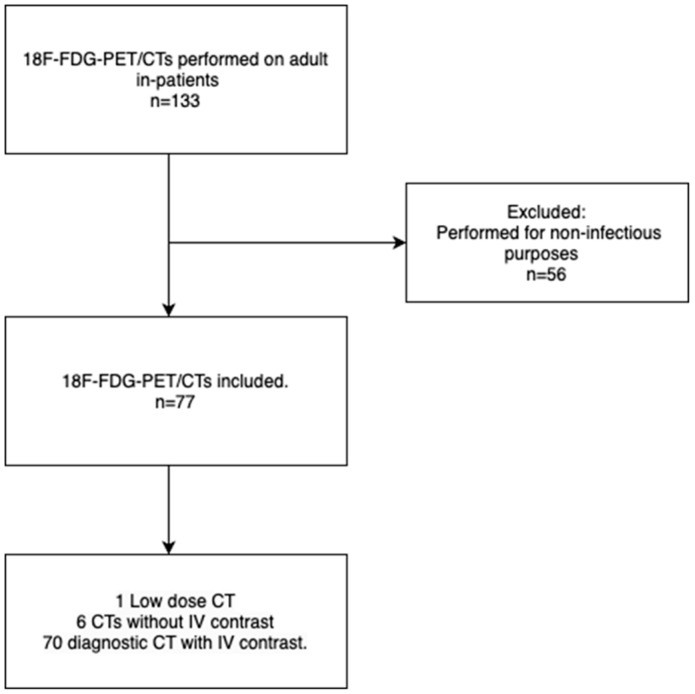
Flowchart depicting the study’s inclusion process.

We identified 133 [^18^F]FDG-PET/CTs performed in search of an infectious focus in patients admitted to the hospital. Of the 133 scans, 56 were excluded because the [^18^F]FDG-PET/CT was performed for purposes unrelated to infection. Thus, 77 cases were included in the study; 70 scans (91%) were performed with contrast-enhanced diagnostic CT, seven were performed as diagnostic CT without contrast due to kidney disease or insufficiency; and only one was performed as low-dose CT. The inclusion process is visualised in Figure 1.

### 3.1. Demographics and Indications

Patient demographics and indications are presented in Table 1.

All parameters recorded can be found in the Supplementary Tables S1 and S2.

### 3.2. Impact of [^18^F]FDG-PET/CT 

An overview of the clinical impact of [^18^F]FDG-PET/CT is presented in Table 2. [^18^F]FDG-PET/CT was considered helpful in the diagnostic process in a total of 47 (61%) cases. Overall, 27 (35%) were true positives, 20 (26%) were true negatives, and [^18^F]FDG-PET/CT led to a direct change in antibiotic management in 20 of 77 (26%) cases. A total of 42 patients (55%) had 72 additional procedures or consults related to [^18^F]FDG-PET/CT findings. 

### 3.3. Subgroup Analysis 

In a subgroup analysis, patients with a previous CT/MRI scan of the thorax/abdomen were compared to those without. We found [^18^F]FDG-PET/CT scans helpful in 25 (69%) cases in the group with previous imaging, compared to 22 (54%) helpful scans in the group with no prior CT imaging. This was not statistically significant (*p* = 0.34). Results from the subgroup analysis are presented in Table 3.

Univariate logistic regression was performed to explore any correlation between true positive cases (dependent variable) and CRP and white blood cell count (WBC) (independent variables). Only WBC reached a statistically significant correlation with a 95% confidence interval (CI) of [1.002–1.285] and *p* = 0.046. Results from the logistic regression are presented in Table 4. 

**Figure 2:**
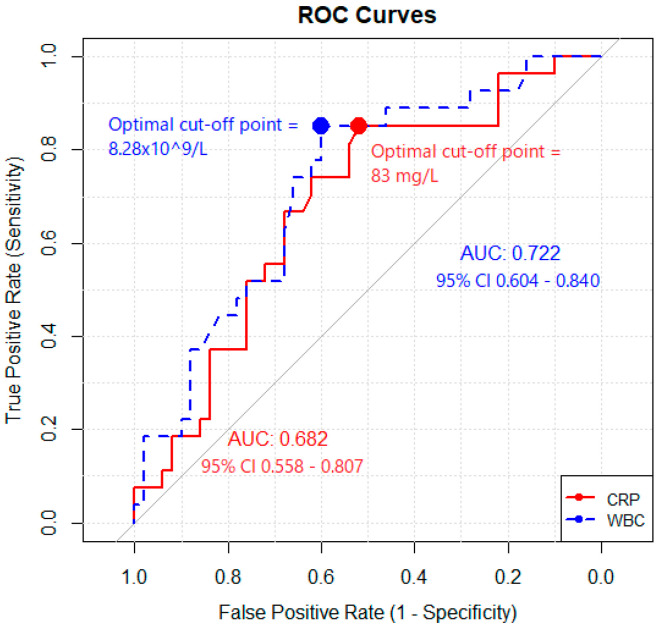
ROC Curve analysis of diagnostic performance and optimal cut-off points for CRP and WBC.

The ROC curve can be seen in Figure 2.

No true positive cases exhibited CRP levels within the normal range; the range of CRP for true positive cases was 11–340 mg/L. Otherwise, CRP showed a moderate diagnostic performance with an AUC of 0.682 with a 95% CI from 0.558 to 0.807. WBC was found to be slightly better, with an acceptable diagnostic performance of 0.722 with a 95% CI of 0.604–0.840. The optimal cut-off point for CRP was 83 mg/L with a sensitivity of 85% and a specificity of 52%. The optimal cut-off point for WBC was 8.28 × 10^9^/L with a sensitivity of 85% and a specificity of 60%. However, from a clinician’s point of view, such cut-off values may be considered speculative and of little value in a clinical reality. 

### 3.4. [^18^F]FDG-PET/CT vs. CT

Of the 27 true positive [^18^F]FDG-PET/CT scans, one was performed without IV contrast. When the CT part was assessed separately, CT alone was able to detect the same pathologies as [^18^F]FDG-PET/CT in 20 out of 29 diagnoses (69%) (Table 5). Thus, [^18^F]FDG-PET/CT could be considered essential in 7/27 (31%) true positive patients, or 7/77 (9%) of all patients.

## 4. Discussion 

### 4.1. Summary of Findings 

This retrospective chart review aimed to evaluate the diagnostic yield of [^18^F]FDG-PET/CT in a broad real-life clinical setting of in-patients with suspected infection or inflammation. In our series, [^18^F]FDG-PET/CT led clinicians to a diagnosis in about 1/3 (35%) of cases while ruling out focal infection in approximately every 1/4 (26%) cases for a total helpfulness in 61% of the patients.

Additionally, we recorded seventy-two additional interventions as a direct result of [^18^F]FDG-PET/CT and found seven incidental diagnoses, including two cancer diagnoses. In approximately 1/4 (26%) cases, [^18^F]FDG-PET/CT led to a change in antibiotic treatment by either extending or reducing duration or changing or stopping treatment. Our regression analysis found increased WBC to be associated with true positive outcome; optimal cut-off point was 8.28 × 10^9^/L to yield a sensitivity of 85% with a specificity of 60%. More importantly, [^18^F]FDG-PET/CT was always negative if CRP levels were normal. In the comparison of [^18^F]FDG-PET/CT and CT, we found that standalone CT detected the same pathologies as [^18^F]FDG-PET/CT in 20/29 (69%) of cases. 

### 4.2. State of the Current Literature 

The available literature on [^18^F]FDG-PET/CT in unclarified infections present considerable challenges: aetiologies are numerous, and populations are heterogeneous due to the non-uniform inclusion and exclusion criteria, high variability of basic characteristics, referring departments, diagnostic protocols, and follow-up [9]. This heterogeneity renders comparisons difficult and impedes our ability to draw firm evidence-based conclusions. Furthermore, the existing literature does not necessarily depict how [^18^F]FDG-PET/CT is used in clinical practice, at least not in our hospital. 

Most studies on [^18^F]FDG-PET/CT in infection investigate a single population, such as Staphylococcus aureus bacteraemia, endocarditis, or FUO. However, very few studies capture the full spectrum of actual use in clinical practice [12]. There is little evidence to support the use of [^18^F]FDG-PET/CTs for patients who do not meet the criteria for FUO or present with bacteraemia. Nonetheless, we frequently encounter these patients in our practice and this specific indication amounted to 27% of cases in our study, despite the lack of scientific support [13]. 

A significant contributor to the heterogeneity of the literature is a significant disparity in how the clinical impact is reported. While some studies report only true positives as “helpful” [9], we have chosen to report “helpful” as both true positive and true negative scans. Whether true negatives should be included in the helpfulness category is a matter of debate. Studies have shown [^18^F]FDG-PET/CT to have a high negative predictive value when it is without findings [14,15], and a meta-analysis suggests that undiagnosed classic FUO patients with negative [^18^F]FDG-PET/CT had a high likelihood of spontaneous remission [15]. However, this is with the benefit of hindsight. To the clinician dealing with an unwell patient, a negative [^18^F]FDG-PET/CT might not offer reassurance and may lead to more testing in actual practice. In contrast to our approach, other studies report detection rate as their primary outcome or use sensitivity/specificity analysis [9,16]. However, this approach is challenging to define due to the multitudes of differential diagnoses that FUO encompasses. Furthermore, some studies evaluate the clinical usefulness of [^18^F]FDG-PET/CT in terms of its impact on patient treatment or management. 

Even though our population does not fit the typical criteria of FUO, our result of 61% helpfulness very closely matches the diagnostic yield found in that population. In a meta-analysis of 16 studies on FUO, the median helpfulness of [^18^F]FDG-PET/CT was 55% [17]. We do not have an explanation for this convergence of results, but we hypothesise that the discretionary judgement of clinicians could be close to as efficient as the stringent criteria of FUO.

The final diagnoses of our patients were predominantly infections. In contrast to our findings, most studies primarily find non-infectious inflammatory diseases, vasculitis, and cancers [18,19,20,21,22]. This difference is most likely attributable to our study setting of in-patients; non-infectious inflammatory diseases are usually managed in outpatient clinic.

A recent study similar to ours by Medvedeva et al. aimed to evaluate the use of [^18^F]FDG-PET/CT in infections in a real-world context [12]. About 40% of their patients were FUO, and 46% were known infections, mostly bacteraemia. Their patients had an average of five radiographic procedures performed before [^18^F]FDG-PET/CT, and they included 61 patients in six years. These differences highlight the earlier use of [^18^F]FDG-PET/CT in our hospital and the extensive prior testing their patients underwent. 

Medvedeva et al. found [^18^F]FDG-PET/CT to have had a clinical impact in 36% of patients, which aligns with our true positive rate of 35%. [^18^F]FDG-PET/CT also led to a change in antibiotic treatment in 25% of patients, closely resembling our findings.

### 4.3. Role of [^18^F]FDG-PET/CT vs. CT in Diagnostic Decision-Making

Unexpectedly, we found a lower percentage of true positives in the group without prior CT/MR imaging. 

A patient group that has undergone less extensive prior testing should yield a higher detection rate [9,23]. We suspect the results were partially influenced by the low true positive rate of endocarditis, which usually proceeds straight to [^18^F]FDG-PET/CT [4]. Of the 19 patients with suspected endocarditis, we could only detect three true positive cases. However, this does not mean [^18^F]FDG-PET/CT has no value in the evaluation of endocarditis. The preliminary workup of endocarditis consists of blood cultures and echocardiography, including TEE [4]. These modalities combined have a good sensitivity regarding native valve endocarditis but fall short in prosthetic valve endocarditis because of artefacts caused by the prosthetic valves [24]. When endocarditis is suspected in patients with artificial heart valves based on positive blood cultures but unconfirmed by echocardiography, [^18^F]FDG-PET/CT becomes crucial. Additionally, [^18^F]FDG-PET/CT can locate or rule out extra-cardiac infections such as spondylodiscitis in high-risk patients [24,25].

When we compared [^18^F]FDG-PET/CT and CT alone, CT alone could indeed have identified a substantial proportion of true positive cases (69%); 15 of the 27 patients had previously undergone CT/MRI, which prompts an interesting question as to why they were subsequently referred for [^18^F]FDG-PET/CT. We propose the following hypotheses to account for this. (1) Disease progression may have occurred between the initial imaging and subsequent [^18^F]FDG-PET/CT examination. This would lead to an overestimation of the benefits of [^18^F]FDG-PET/CT. In diseases like spondylodiscitis, MRI has a significantly lower sensitivity than [^18^F]FDG-PET/CT in the first 14 days of the disease, as [^18^F]FDG-PET/CT can detect functional abnormalities which precede morphologic changes [26,27]. It would have been interesting to examine the time period between scans, but this information was not included in our data extraction. (2) In some cases, [^18^F]FDG-PET/CT was probably used to confirm findings seen on previous imaging. This would partly explain the higher true positive rate of those with previous imaging, but we did not explore previous imaging reports in this study as it was beyond the scope of this study. (3) [^18^F]FDG-PET/CT might have been used defensively to rule out further disease foci. The additional metabolic information provided by the PET component may affirm that pathology is indeed isolated and alleviate concerns about other suspected conditions. (4) Despite being blinded to the PET images, the radiologist was aware that patients had undergone [^18^F]FDG-PET/CT. This knowledge could have subtly influenced their interpretation of the CT scans. Knowing that a patient had been referred for an [^18^F]FDG-PET/CT might have led them to re-evaluate certain findings, which might otherwise have been dismissed as normal, insignificant, or equivocal. For example, the radiologist might typically have attributed observed changes in the spine to osteoarthritis, but with the knowledge of the [^18^F]FDG-PET/CT referral, they might lean towards a diagnosis of spondylodiscitis. 

### 4.4. Non-Standardised Testing and Physician Discretion in [^18^F]FDG-PET/CT Utilisation

In comparison to other studies, our patient population was not required to undergo a standardised testing protocol before referral to [^18^F]FDG-PET/CT. This potentially limits comparisons to studies with highly selected patient groups with strict requirements for obtaining an [^18^F]FDG-PET/CT scan. Additionally, combined with Denmark’s single-payer healthcare system, the use of [^18^F]FDG-PET/CT was entirely at the treating physician’s discretion and not limited by insurance-based restrictions. Currently, there are no established official national or international guidelines for when to obtain an [^18^F]FDG-PET/CT scan in the workup of FUO or bacteraemia. [^18^F]FDG-PET/CT is usually performed as a second-line examination after routine workup has turned up negative [18,28], which could cause patients to undergo additional procedures that may not yield significant findings. However, if [^18^F]FDG-PET/CT is performed earlier in the workup, it will diagnose patients who may otherwise be diagnosed by other means or by self-limiting disease. This effect was demonstrated in a study using [^18^F]FDG-PET/CT as first-line imaging in an entirely unselected population. In this study, Ferda et al. reported a helpfulness of 92% [23]. Therefore, comparisons should be cautiously interpreted due to their heterogeneity.

### 4.5. Managing Incidental Findings in [^18^F]FDG-PET/CT: A Clinical Dilemma

Incidental findings by [^18^F]FDG PET/CT are relatively common and represent a clinical challenge in cancer patients as well as inflammatory settings [29,30,31,32]. There is a fine line between false positive findings and those potentially cancerous in nature. The former are of benign aetiology and would not have caused any issues for the patient if left undetected or simply represent physiologic tracer uptake in healthy tissue or artefacts. These incidental findings may lead to a potential waste of healthcare resources if they are further explored. The latter lesions may actually benefit the patients as early detection can increase the chances of successful treatment [33]. In this study, we reported a high number of additional investigations into these incidental findings. Substantial evidence supports the investigation of certain incidental findings, such as the colon [34] and thyroid [35], but most incidental findings are left to interpretation. 

### 4.6. Predictors of [^18^F]FDG-PET/CT Outcome

Several laboratory tests have been examined as predictors of usefulness of [^18^F]FDG-PET/CT in bacteraemia and FUO. CRP, WBC, and erythrocyte sedimentation rate (ESR) are the most well-examined [9]. No exact cut-off value has been established to stratify patients in either FUO or bacteraemia, but, generally, CRP is considered the most significant. However, the association remains inconclusive and controversial [22,36]; some studies found CRP to be an independent predictor for the outcome of [^18^F]FDG-PET/CT [19,37,38] while others found no such correlation [21,39]. Perhaps the best predictive value of CRP is when it is normal; several studies found [^18^F]FDG-PET/CT to be unhelpful if CRP was normal [22,37,40]. In regression analysis of our study, we were unable to establish a significant association for CRP as a predictor of outcome, but no true positive case exhibited normal CRP levels. This corroborates the existing literature that [^18^F]FDG-PET/CT is of limited value in FUO when CRP levels are normal. However, WBC did reach statistical significance. It is, however, important to bear in mind that while laboratory measures and cut-off values can offer guidance, they are largely arbitrary and do not directly translate into clinical practice.

### 4.7. Limitations

Our study has the inherent limitations of a single-centre retrospective study, which limits the generalisability of our findings. Patient referrals were heterogeneous by design, and the patient group’s composition differs significantly from other published studies that mainly focus on specific indications. Due to Danish law prohibiting access to the database of patients’ complete medical journals for research, we were limited to The Region of Southern Denmark’s Electronic Patient Journal (EPJ), which was introduced at the start of 2022. This limited our access to past health information not mentioned in the new system and some data were missing due to incomplete migration of older data to the new system. 

## 5. Conclusions 

The present study is one of the first studies investigating [^18^F]FDG-PET/CT in a broad and clinically unselected population of in-patients with suspected infection or inflammation as opposed to studies with more stringent FUO-criteria. We found [^18^F]FDG-PET/CT helpful in the workup of infection in 61% of cases, closely resembling results in classic FUO-studies, despite the differences in population. 

We found that incidental findings on [^18^F]FDG-PET/CT led to almost one extra examination per patient with relatively few clinically relevant findings. 

CT could detect the same true positive pathologies as [^18^F]FDG-PET/CT in a substantial number of cases; the pathologies exclusively detected by [^18^F]FDG-PET/CT were endocarditis and spondylodiscitis. 

While our findings do not conclusively suggest that [^18^F]FDG-PET/CT scans are being utilised prematurely, results point to their predominant use in ruling out infection instead of confirming it.

Results from further prospective studies are needed to evaluate the benefit of early [^18^F]FDG-PET/CT in in-patients and to corroborate our findings. Furthermore, the economics of the early introduction of [^18^F]FDG-PET/CT in in-patients and the emergency department remains virtually unexplored. 

As of now, no guidelines exist for the initial workup and imaging procedures that should be undertaken before [^18^F]FDG-PET/CT scanning. Further research could focus on establishing guidelines to help the diagnostic process.

## Figures and Tables

**Table 1:** Baseline characteristics, laboratory values, and indications of the 77 included participants.

Patient Characteristics	*n*	%/Range
**Sex**		
Male	53	69%
Female	24	31%
Mean age	68	21–94
**Dead at follow-up**		
Yes	16	21%
No	61	79%
**Referring department**		
Department of Nephrology	19	24%
Department of Cardiology	17	22%
Department of Internal medicine	15	19%
Emergency Department	13	17%
Department of Respiratory Diseases	9	11%
Department of Orthopaedic Surgery	2	3%
Department of General Surgery	1	1%
Intensive Care Unit (ICU)	1	1%
**Days of admission before PET/CT was ordered**		
Mean	8	0–74
**White blood cell count**		
Leucocytosis > 8.8 × 10^9^/L	42	55%
Mean	10.4	5.4–25.5
**C-reactive protein**		
>10 mg/L	72	93%
<10 mg/L	5	7%
Mean	120	1–340
**Indications**	*n*	%/mean
**Stated indication from referring clinician**		
Unknown infectious foci with positive culture(s)	24	31%
Unknown infectious foci without positive culture(s)	21	27%
Evaluation of endocarditis	19	25%
Spondylodiscitis	7	9%
Other	6	8%
**Any positive cultures?**		
Yes	49	63%
No	28	36%
**Imaging prior to [^18^F]FDG-PET/CT**		
None	2	3%
Chest X-ray	59	77%
CT thorax/abdomen	34	44%
Transoesophageal Echocardiography (TEE)	34	44%
Ultrasound	14	18%
X-ray of the skeleton	12	16%
CT cerebrum	6	8%
MR of the skeleton	5	6%
Other	9	12%
**Total**	175	2.2 ^a^

*n* = number of patients. ^a^ = mean.

**Table 2:** Helpfulness and impact of [^18^F]FDG-PET/CT on treatment, additional examinations incidental findings, outcomes, and final diagnoses.

Impact of [^18^F]FDG-PET/CT	*n*	%
**Was the scan considered helpful?**		
True positive	27	35%
True negative	20	26%
Helpful total	47	61%
Unhelpful	30	39%
**Did [^18^F]FDG-PET/CT lead to a change in antibiotics?**		
Change	20	26%
No change	57	74%
**Additional procedures/consultations following 72 [^18^F]FDG-PET/CT findings in 42 patients (55%)**		
Specialist consultation	30	39%
Colonoscopy	8	10%
MR	7	9%
Cancer fast track	7	9%
Other	20	-
**Did incidental findings lead to a diagnosis?**		
Non-cancerous	5	6%
Cancerous	2	3%
No	70	91%
**Final Diagnosis**		
Infection without a known agent	15	19%
Endocarditis	14	18%
Pneumonia	11	14%
Spondylodiscitis	9	12%
Bacteraemia	7	9%
Urinary tract infection (UTI)	6	8%
Abscess	4	6%
Pericarditis	3	4%
Other	11	14%
Total	80 ^a^	100%

*n* = number of patients. ^a^ = Three patients were diagnosed with two infections and have been counted twice. One patient was diagnosed with spondylodiscitis and endocarditis, one patient with UTI and pneumonia, and one patient with pneumonia and spondylodiscitis. One patient can receive several consultations. Consultations are counted separately from other examinations, i.e., a colonoscopy does not count as a consultation.

**Table 3:** Subgroup analysis comparing the usefulness of [^18^F]FDG-PET/CT in patients with and without prior CT/MR imaging.

	Prior CT/MRI	No Prior CT/MRI	Total
**True positive**	15 (42%)	12 (29%)	27
**True Negative**	10 (28%)	10 (24%)	20
**Helpful total**	25 (69%)	22 (54%)	47
**Unhelpful**	11 (31%)	19 (46%)	30
**Total**	36 (100%)	41 (100%)	Total number of patients 77

χ^2^ (1, N = 77) = 2.5, *p* = 0.34.

**Table 4:** Results of the univariate logistic regression.

Variable	Coefficient	Standard Error	Odds Ratio	95% Confidence Interval	*p*-Value
**CRP**	0.004	0.003	1.004	0.997–1.001	0.285
**WBC**	0.126	0.063	1.135	1.002–1.285	0.046

**Table 5:** Comparative analysis of pathologies missed and matched by CT alone.

		*n*
**Diagnosis missed by CT**	Spondylodiscitis	5
	Endocarditis	3
	Wound infection	1
	**Total**	**9**
**Diagnosis matched by CT**	Pneumonia	8
	Abscess	4
	Spondylodiscitis	2
	Pericarditis	2
	Sacroiliitis	1
	Endocarditis	1
	Prosthetic hip infection	1
	Cholecystitis	1
	Pyometrium	1
	**Total**	**20**

*n* = Number of diagnoses. One patient presented with both endocarditis and spondylodiscitis. One patient presented with spondylodiscitis and pneumonia.

## Data Availability

Data is not readily available due to Danish GDPR legislation.

## Supplementary Materials

The following supporting information can be downloaded at: https://www.mdpi.com/article/10.3390/diagnostics14131420/s1, Table S1: Baseline Characteristics and Laboratory values of the 77 included participants. Table S2: Summary of Clinical Symptoms, Cultures, and Pre-PET/CT Imaging. Table S3: Impact and Outcomes of 18F-FDG-PET/CT on Treatment, Additional Examinations, Incidental Findings, and Final Diagnoses.
