# Supplementary material for: The Diagnostic Yield of [18F]FDG-PET/CT in a Heterogeneous In-Patient Population with Suspected Infection or Inflammation Is Comparable to Findings in Patients with Classic Fever of Unknown Origin
Source: Diagnostics (Basel). 2024 Jul 3;14(13):1420. doi: 10.3390/diagnostics14131420 (PMC11241151; doi:10.3390/diagnostics14131420)
Supplement: Supplementary file 1 [file diagnostics-14-01420-s001.zip › diagnostics-3028993-supplementary.pdf]

Supplementary Table 1: Baseline Characteristics and Laboratory values of the 77 included participants

Patient characteristics	n	% / range
**Sex**		
Male	53	69%
Female	24	31%
Median age	74	21-94 years
Mean age	68	IQR 22[b]
**Dead at follow-up**		
Yes	16	21%
No	61	79%
**Referring department**		
Department of Nephrology	19	24%
Department of Cardiology	17	22%
Internal medicine	15	19%
Emergency Department	13	17%
Department of Respiratory Diseases	9	11%
Department of Orthopaedic Surgery	2	3%
Department of General Surgery	1	1%
Intensive care unit (ICU)	1	1%
**Days of admission before PET/CT was ordered**		
Mean	8	0-74 days
Median	5	IQR 4[b]
**Immunocompromising factors**		
Patients with one or more immunocompromising factors	15	19%
Active malignancy or Chemotherapy	4	5%
Prednisolone > 10 mg/day	6	8%
Prednisolone < 10 mg/day	3	4%
Organ transplant	2	3%
Immunosuppressive /modulating	4	5%
**Diabetes**		
Yes	23	30%
No	54	70%
**Comorbidities**		
Patients with one or more morbidities	34	44%
Kidney failure	20	26%
Chronic obstructive pulmonary disease/ fibrosis	17	22%
Heart failure	15	19%
Liver cirrhosis	1	1%
**Foreign bodies**		
Patients with one or more foreign bodies	38	49%
Pacemaker	17	22%
Prosthetic heart valve	15	19%
Alloplasty	11	14%
JJ catheter	3	4%
Spinal osteosynthesis material	2	3%
**Fever**		
Yes	16	21%
No	61	79%
**White blood cell count**		
Leucocytosis > 8.8 mg/mL	42	55%
Mean	10.4	5.4-25.5[a]
Median	9.6	IQR 6.6[b]
**C-reactive protein**		
>10 mg/L	72	93%
< 10 mg/L	5	7%
Mean	120	1-340[a]
Median	106	IQR 130[b]

n= number of patients.

[a]=Range . [b]=Interquartile range

Supplementary Table 2: Summary of Clinical Symptoms, Cultures, and Pre-PET/CT Imaging.

Indications	n	% / mean
**Symptoms of infection**		
One or more symptoms of infection	43	56%
Wounds/skin infection	13	17%
Back or neck pain	12	16%
Abdomen pain	10	13%
Joint pain	3	4%
Flank pain	3	4%
Chest Pain	2	3%
Pain in joint with alloplasty	2	3%
Pain when urinating	2	3%
Groin pain	1	1%
**Stated indication from referring clinician**		
Unknown infection foci with positive culture(s)	24	31%
Unknown infection foci without positive culture	21	27%
Evaluation of endocarditis	19	25%
Spondylodiscitis	7	9%
Prosthetic infection	2	3%
Fever of unknown ethiology (FUE)	1	1%
Epidural abscess	1	1%
Renal failure	1	1%
Psoas Abscess	1	1%
**Any positive cultures?**		
Yes	49	63%
No	28	36%
**Cultures taken**		
None	0	
Blood	75	97%
Urine	66	86%
Airways	63	82%
Feces	11	14%
Wound swab	4	5%
Tissue sample	3	4%
Spinal fluid	3	4%
Abscess	1	1%
Ascites	1	1%
Pleural fluid	1	1%
**Imaging prior to 18F-FDG-PET/CT**		
None	2	3%
Chest x-ray	59	77%
CT thorax/abdomen	34	44%
Transesophageal echocardiography (TEE)	34	44%
Ultrasound	14	18%
X-ray of the skeleton	12	16%
CT cerebrum	6	8%
MR of the skeleton	5	6%
Renography	3	4%
Single-photon emission computed tomography (SPECT)	2	3%%
Magnetic resonance cholangio pancreatography (MRCP)	1	1%
18F-FDG-PET/CT	1	1%
CT sinus	1	1%
MR abdomen	1	1%
Total	175	2,2[c]

Table 2: Indications for 18F-FDG-PET/CT

n= number of patients.

[c]*=mean*

Supplementary Table 3: Impact and Outcomes of 18F-FDG-PET/CT on Treatment, Additional Examinations, Incidental Findings, and Final Diagnoses

Impact of 18F-FDG-PET/CT	n	%
**Was scan considered helpful?**		
True positive	27	35%
True negative	20	26%
Helpful total	47	61%
Unhelpful	30	39%
**Did 18F-FDG-PET/CT lead to a change in antibiotics?**		
Change	20	26%
No change	57	74%
**Additional examinations as a result of 18F-FDG-PET/CT findings**		
No further testing	35	45%
Consultation	30	-
Colonoscopy	8	10%
MR	7	9%
Cancer fast track	7	9%
Biopsy	3	4%
Abscess drainage	3	4%
Joint puncture	3	4%
Transesophageal echocardiography (TEE)	3	4%
Operation	2	3%
CT	2	3%
X-ray of skeleton	1	1%
Cystoscopy	1	1%
Ultrasound	1	1%
Magnetic resonance cholangio pancreatography (MRCP)	1	1%
Total	72	-
**Did incidental findings lead to a diagnosis**		
Yes	7	9%
No	70	91%
**Incidental diagnoses**		
Cholestasis	1	
Thyroid tumour	1	
Uterus fibroma	1	
Colon cancer	1	
Benign rectum tumour	1	
Necrosis of the femoral head	1	
Non-alcoholic fatty liver disease 9b	1	
**Final diagnosis**		
Infection without a known agent	15	19%
Endocarditis	14	18%
Pneumonia	11	14%
Spondylodiscitis	9	12%
Bacteraemia	7	9%
Urinary tract infection (UTI)	6	8%
Abscess	4	6%
Pericarditis	3	4%
Infectious sacroiliitis	1	1%
Pneumocystis	1	1%
Nephrotic syndrome	1	1%
Prostetic hip infection	1	1%
Wound infection	1	1%
Myelomatosis	1	1%
Pyometrium	1	1%
Erysipelas	1	1%
Unknown non-infectious etiology	1	1%
Acute renal failure without known etiology	1	1%
Myelodysplastic syndrome	1	1%
Total	80[a]	100%

n= number of patients
[a]= Three patients were diagnosed with two infections and have been counted twice.
One patient was diagnosed with Spondylodiscitis and endocarditis, one patient with UTI and pneumonia, and one patient with pneumonia and spondylodiscitis.
One patient can receive several consultations.
Consultations are counted separately from other examinations, I.e. a colonoscopy does not count as a consultation.
